# *In silico* Screening and Heterologous Expression of a Polyethylene Terephthalate Hydrolase (PETase)-Like Enzyme (SM14est) With Polycaprolactone (PCL)-Degrading Activity, From the Marine Sponge-Derived Strain *Streptomyces* sp. SM14

**DOI:** 10.3389/fmicb.2019.02187

**Published:** 2019-10-01

**Authors:** Eduardo L. Almeida, Andrés Felipe Carrillo Rincón, Stephen A. Jackson, Alan D. W. Dobson

**Affiliations:** ^1^School of Microbiology, University College Cork, Cork, Ireland; ^2^Environmental Research Institute, University College Cork, Cork, Ireland

**Keywords:** synthetic polyesters, polycaprolactone, PETases, *Streptomyces*, plastics

## Abstract

Plastics, such as the polyethylene terephthalate (PET), are widely used for various industrial applications, due to their physicochemical properties which are particularly useful in the packaging industry. However, due to improper plastic waste management and difficulties in recycling, post-consumer plastic waste has become a pressing issue for both the environment and for human health. Hence, novel technologies and methods of processing plastic waste are required to address these issues. Enzymatic-assisted hydrolysis of synthetic polymers has been proposed as a potentially more efficient and environment-friendly alternative to the currently employed methods. Recently, a number of PET hydrolases have been described, and in particular a PETase derived from *Ideonella sakaiensis* 201-F6 (IsPETase), which appears to be the most efficient and substrate-specific bacterial PET hydrolase enzyme discovered to date. In order to further investigate this class of PETase-like enzymes, we employed an *in silico-*based screening approach on the biotechnologically relevant genus *Streptomyces*, including terrestrial and marine isolates; in a search for potential PETase homologs. From a total of 52 genomes analyzed, we were able to identify three potential PETase-like enzymes, all of which were derived from marine-sponge associated *Streptomyces* isolates. A candidate PETase-like gene (SM14est) was identified in *Streptomyces* sp. SM14. Further *in silico* characterization of the SM14est protein sequence and its predicted three-dimensional structure were performed and compared to the well-characterized IsPETase. Both the serine hydrolase motif Gly-x1-Ser-x2-Gly and the catalytic triad Ser, Asp, His are conserved in both sequences. Molecular docking experiments indicated that the SM14est enzyme possessed the capacity to bind plastics as substrates. Finally, polyesterase activity was confirmed using a polycaprolactone (PCL) plate clearing assay which is a model substrate for the degradation of plastics; following heterologous expression of SM14est in *Escherichia coli*, with secretion being facilitated by the native *Streptomyces* signal peptide. These findings provide further insights into this important class of PETase-like enzymes.

## Introduction

Plastics are materials that have been produced on a large scale from the 1950s onwards, and since then have been widely used for various applications, and have become almost indispensable in modern society (Jambeck et al., 2015; Geyer et al., 2017; Lebreton and Andrady, 2019). In the 1960s, plastics accounted for less than 1% of municipal solid waste in the United States, but steadily increased to around 10% by 2005 in countries with middle to high income (Jambeck et al., 2015; Geyer et al., 2017). This was largely due to their advantageous properties, such as their low production cost and bio-inertia, which are particularly useful for the packaging industry, when compared to other materials. This has resulted in the use of plastics in the packaging sector, which accounts for around 40% of the plastic converter demand in Europe (PlasticsEurope, 2018; Lebreton and Andrady, 2019). However, some of these aforementioned characteristics have resulted in plastics becoming a critical problem from an environmental perspective; as many synthetic plastics are highly recalcitrant to biodegradation and can persist for long periods of time in the environment (Wei and Zimmermann, 2017b).

In 2017, there was an estimated worldwide plastics production of 348 million tons – an increase from the 335 million tons estimated for the previous year, and this does not include polyethylene terephthalate (PET)-, polyamide (PA)-, and polyacryl-fibers (PlasticsEurope, 2018). At the current rate, this number is expected to double in the next 20 years (Lebreton and Andrady, 2019). In the past decade, plastic waste management policies have helped considerably in reducing post-consumer plastic waste being disposed into the environment. For example, in Europe more collected plastic waste (31.1% of 27.1 million tons of collected plastic waste in 2016) was submitted to recycling rather than to landfills, for the first time. However, landfills and incineration for energy recovery still account for 27.3 and 41.6% of the collected plastic post-consumer waste, respectively (PlasticsEurope, 2018).

Notwithstanding this, the aforementioned metrics do not take into account global mismanaged plastic waste which enters the natural environment, at locations others than landfills. A recent study has estimated that between 60 and 99 million metric tons of mismanaged plastic waste was produced globally in 2015, and that this number could triple by 2060 (Lebreton and Andrady, 2019). Mismanaged plastic waste is particularly concerning when it effects the marine environment. It has been calculated that in 2010 between 4.8 and 12.7 million metric tons of plastic waste entered our oceans, and this data only accounted for coastal countries (Jambeck et al., 2015). Sunlight and other weathering effects cause the fragmentation of plastic debris into milli- and micro-metric particles (<5 mm), which are defined as micro-plastics (Geyer et al., 2017; Lebreton and Andrady, 2019). These micro-plastics are now believed to be ubiquitous in soil and aquatic environments, and are commonly ingested by animals (Santillo et al., 2017; Lebreton and Andrady, 2019). This is especially concerning, since micro-plastics can adsorb and concentrate pollutants present in the ocean and transfer them along the food chain, particularly to seafood species that are consumed by humans (Santillo et al., 2017). Highlighting the issue of the ubiquitous presence of micro-plastics in the marine environment, a recent study detected the ingestion of micro-plastics by deep-sea amphipods, at depths ranging from 7,000 to 10,890 m (Jamieson et al., 2019). Additionally, the deepest ever sub diving recorded to date has registered the presence of plastics on the ocean floor, at a depth of 10,927 m (Street, 2019). It is alarming to find plastics, which are materials with a history of less than a century of large-scale production, already being so widespread in nature; with the potential for extensive negative impacts, many of which have yet to be fully realized. Hence, better plastic waste management and processing solutions are urgently required.

Currently, the majority of plastic waste recycling is based on mechanical recycling (collection, sorting, washing, and grinding) (Ragaert et al., 2017). However, the presence of organic and inorganic impurities in post-consumer plastic waste presents a huge challenge for mechanical recycling (Drzyzga and Prieto, 2019). On the other hand, chemical recycling has been applied as an alternative for improved plastic waste management processes, in which the plastic polymers can be converted into raw materials that can be used for the synthesis of chemicals, fuels, or virgin plastics (Drzyzga and Prieto, 2019). Strictly chemical methods, however, require the use of toxic chemicals and high temperatures, and can also be quite costly (Awaja and Pavel, 2005; Wei and Zimmermann, 2017a). Therefore, enzymatic hydrolysis of synthetic polyesters has been proposed as a potentially more efficient and environment friendly method for the recycling of plastic waste (Wei and Zimmermann, 2017a; Drzyzga and Prieto, 2019).

In the past decade, a number of bacterial enzymes capable of degrading synthetic polyesters, including the widely used PET, have been identified (Wei and Zimmermann, 2017a; Kawai et al., 2019). These enzymes are commonly classified as members of the cutinase, lipase and esterase classes of enzymes, and to date have mainly been identified in thermophilic actinomycetes, particularly in the genus *Thermobifida* (Silva et al., 2011; Wei and Zimmermann, 2017a). More recently, Yoshida et al. (2016) isolated a bacterium from a PET plastic bottle recycling plant in Sakai, Japan, that was capable of degrading and assimilating PET as its major energy and carbon source – namely *Ideonella sakaiensis* 201-F6. The protein identified as being responsible for the hydrolysis of PET (ISF6_4831) was then defined as a PETase (or PET hydrolase) enzyme (EC 3.1.1.101) (Yoshida et al., 2016). The PETase from *Ideonella sakaiensis* 201-F6 has been shown to possess a relatively higher enzymatic activity and substrate specificity for PET than other previously described PET hydrolases, in addition to the ability to degrade PET at moderate temperatures (around 30°C) (Yoshida et al., 2016; Joo et al., 2018). Since then, a number of studies have been undertaken in a concerted effort to characterize this enzyme and the underlying metabolic and biochemical processes involved in the degradation of PET (Han et al., 2017; Chen et al., 2018; Joo et al., 2018; Liu et al., 2018).

The *Streptomyces* genus, member of the Actinomycetales order, is well-known to produce compounds and enzymes of industrial and clinical interest, particularly antibiotics, for which it is considered the largest producer in the microbial world (Watve et al., 2001; Hwang et al., 2014; Ser et al., 2017; Spasic et al., 2018). Recent efforts to exploit the biotechnological potential of *Streptomyces* species have largely focused on the identification of bioactive small molecules and secondary metabolites biosynthetic gene clusters (Chevrette et al., 2019; Manteca and Yagüe, 2019). In this respect, the focus has started to shift toward *Streptomyces* isolates derived from varied niche environments, such as those isolated from the marine environment, which are still not well-characterized and majorly unexplored organisms, when compared to the previously more commonly studied soil-derived isolates (Dharmaraj, 2010; Hassan et al., 2017; Jin et al., 2018; Xu et al., 2018; Almeida et al., 2019).

*Streptomyces* isolates from soil ecosystems have also been studied for their synthetic polyesters-degrading capabilities (Calabia and Tokiwa, 2004; Shivlata and Satyanarayana, 2015). These include *Streptomyces* sp. strain MG (Tokiwa and Calabia, 2004) and *Streptomyces thermoviolaceus* (Chua et al., 2013), which can degrade polycaprolactone (PCL); together with *Streptomyces bangladeshensis* 77T-4, which degrades poly(D-3-hydroxybutyrate) (PHB) (Hsu et al., 2012). Given that marine *Streptomyces* sp. SNG9 had previously been reported to degrade PHB (Mabrouk and Sabry, 2001), coupled with the fact that marine *Streptomyces* isolates are likely to have been exposed to plastics and/or microplastics in marine ecosystems – in particular those isolates which are associated with marine sponges (phylum *Porifera*), which filter large quantities of seawater (up to 24,000 L of water per day/Kg sponge) on a daily basis to obtain nutrients (Taylor et al., 2007; Food and Agriculture Organization of the United Nations, 2017; Godefroy et al., 2019) – we reasoned that marine sponge-derived *Streptomyces* species may possess enzymes with an ability to degrade synthetic polymers. In this study, we screened a number of *Streptomyces* species, including both terrestrial and marine-derived isolates, using an *in silico*-based analysis to interrogate their genomes for potential PETase homologs. A candidate PETase-like gene was identified in *Streptomyces* sp. SM14 and enzyme activity was confirmed following heterologous expression of this gene in *Escherichia coli*. This is the first report of a PETase-like enzyme being identified in a marine sponge-derived *Streptomyces* spp. isolate, and we believe that this study provides further insights into our current knowledge of this important class of enzymes.

## Materials and Methods

### Data Sets

The reference data set was comprised of 15 amino acid sequences of enzymes with previously demonstrated synthetic polyesters-degrading capabilities (Table 1) (Yoshida et al., 2016; Wei and Zimmermann, 2017a; Danso et al., 2018; Joo et al., 2018; Kawai et al., 2019). A lipase from *Streptomyces exfoliatus* (PDB ID: 1JFR), which is a cutinase-like enzyme (Wei et al., 1998; Kawai et al., 2019), was also included in the reference data set (Table 1), which although not possessing demonstrated polyester-degrading activity, served as an outgroup for the subsequent *in silico* analyses.

**TABLE 1:** Reference data set comprising of 15 PETase-like enzymes with demonstrated PET-degrading activity, including the ISF6_483 protein from *Ideonella sakaiensis* strain 201-F6 (IsPETase), and additionally the cutinase-like lipase from *Streptomyces exfoliatus* (PDB ID: 1JFR).

**Gene**	**Source**	**UniProt**	**GenBank**	**References**
**name**	**accession**	**accession**		
(ISF6_483 IsPETase)	*Ideonella sakaiensis* strain 201-F6	A0A0K8P6T7	GAP38373	Yoshida et al., 2016
Cut190	*Saccharomonospora viridis*	W0TJ64	BAO42836	Kawai et al., 2014
Tcur_1278	*Thermomonospora curvata* DSM 43183	D1A9G5	ACY96861	Chertkov et al., 2011; Wei et al., 2014
Tha_Cut1	*Thermobifida alba*	E9LVH7	ADV92525	Ribitsch et al., 2012a
Thh_Est	*Thermobifida halotolerans*	H6WX58	AFA45122	Ribitsch et al., 2012b
Thc_Cut1	*Thermobifida cellulosilytica*	E9LVH8	ADV92526	Herrero Acero et al., 2011
Thc_Cut2	*Thermobifida cellulosilytica*	E9LVH9	ADV92527	Herrero Acero et al., 2011
Thf42_Cut1	*Thermobifida fusca*	E9LVI0	ADV92528	Herrero Acero et al., 2011
cut-1.KW3	*Thermobifida fusca*	E5BBQ2	CBY05529	Herrero Acero et al., 2011
cut-2.KW3	*Thermobifida fusca*	E5BBQ3	CBY05530	Herrero Acero et al., 2011
LCC	Leaf-branch compost metagenome	G9BY57	AEV21261	Sulaiman et al., 2012
cut_1	*Thermobifida fusca*	G8GER6	AET05798	Hegde and Veeranki, 2013
cut_2	*Thermobifida fusca*	Q6A0I4	AET05799	Hegde and Veeranki, 2013
Tfu_0882	*Thermobifida fusca* XY	Q47RJ7	AAZ54920	Chen et al., 2010
Tfu_0883	*Thermobifida fusca* XY	Q47RJ6	AAZ54921	Chen et al., 2010
Lipase (1JFR)	*Streptomyces exfoliatus*	Q56008	AAB51445	Wei et al., 1998

The *Streptomyces* genomes data set comprised of 52 *Streptomyces* genome sequences obtained from GenBank (Benson et al., 2018), including 23 genomes from terrestrial isolates, and 29 from marine isolates (Supplementary Table S1). Open reading frames (ORFs) and their respective translated amino acid sequences were obtained using Prokka (Seemann, 2014).

### Bacterial Strains

*Streptomyces* strain SM14 was isolated from the sponge *Haliclona simulans* (class *Demospongiae*, order *Haplosclerida*, family *Chalinidae*) which was sampled by SCUBA diving at a depth of 15 m in Kilkieran Bay, Galway, Ireland (N 53°18′56.6′′, W 09°40′08.4′′) as previously described (Kennedy et al., 2009). The NEB^®^ 5-alpha and the BL21(DE3) competent *E. coli* cells were obtained from New England Biolabs, Inc., United States.

### Protein Homology Search and Phylogeny Analysis

Potential PETase-like proteins were identified in the *Streptomyces* genomes data set by performing an homology search using BLASTP (e-value threshold of 1e-30, maximum subject sequence length of 400 aa) (Altschul et al., 1990; Camacho et al., 2009). Protein alignments were performed using Muscle (Edgar, 2004), and phylogeny analysis was performed using MEGA X (maximum likelihood statistical method; 500 bootstrap replications; 50% bootstrap cut-off value; LG + G + F model) (Kumar et al., 2018).

### PCL Plate Clearing Assay

Polycaprolactone (PCL) plate clearing assays were performed based on previously described studies (Nishida and Tokiwa, 1993; Murphy et al., 1996; Nishida et al., 1998; Nawaz et al., 2015). PCL is a synthetic polyester that has previously been used as a model substrate to assess both PETase and cutinase enzymatic activities (Nyyssölä et al., 2013; Danso et al., 2018). PCL with an average molecular weight of 80,000 was used (Sigma-Aldrich^®^). PCL emulsion was prepared with 1% m/v of PCL in acetone, at 50°C with magnetic stirring. Water, agar (1.5% m/v) and LB medium (2% m/v) were added to the emulsion, at 50°C with magnetic stirring until the acetone evaporated. The medium was then autoclaved and poured into plates. *Streptomyces* sp. SM14 and transformed *E. coli* BL21(DE3) containing the pET20b:SM14est vector construct were inoculated onto the plates and incubated at 28°C for up to 12 days. For enzyme activity assessment using *E. coli* as the heterologous host, isopropyl-β-D-thiogalactopyranoside (IPTG) was added to the medium at a concentration of 0.5 mM and plates were incubated at 28°C for up to 4 days. As a negative control, *E. coli* BL21(DE3) containing the pET-20b(+) plasmid without the insert did not show any PCL-degrading activity (results not shown). It has recently been reported that *E. coli* BL21(DE3) can be employed as a host system in screens for polyesterase activity, as it does not possess PCL-degrading capabilities (Molitor et al., 2019). Additionally, 12 other marine sponge-derived isolates were also assayed for polyesterase activity using the PCL plate clearing assay. SM1, SM3, SM4, SM7, SM8, SM9, SM11, SM13, SM17, and FMC008 which had previously been isolated from the marine sponge *Haliclona simulans* Kennedy et al., 2009), together with B188M101 and B226SN101 isolated from the deep sea sponges *Lissodendoryx diversichela* and *Inflatella pellicula* respectively (Jackson et al., 2018); were grown in LB medium + 1% PCL emulsion at 28°C for 12 days (data not shown).

### Protein Structure Analysis, Modeling, and Molecular Docking

Amino acid sequence analysis was performed and graphically represented using ESPript 3.0 (Robert and Gouet, 2014). *In silico* protein structure prediction was performed using the SWISS-DOCK webserver (Guex et al., 2009; Benkert et al., 2011; Bertoni et al., 2017; Bienert et al., 2017; Waterhouse et al., 2018), and the UCSF Chimera software was used for structure analysis and three-dimensional model rendering (Pettersen et al., 2004). Molecular docking experiments were performed using AutoDock Vina, MGLtools^1^, AutoDockTools (ADT) and UCSF Chimera (Sanner, 1999; Pettersen et al., 2004; Trott and Olson, 2009), with the model substrate BHET [*Bis*(2-hydroxyethyl) terephthalate, Zinc database ID: ZINC02040111] molecule as the ligand (Irwin and Shoichet, 2005; Irwin et al., 2012). BHET has previously been used as a model substrate for PET degradation both *in vitro* and *in silico* through molecular docking studies (Hantani et al., 2018; Joo et al., 2018; Liu et al., 2018).

### Heterologous Expression

**FIGURE 1:**
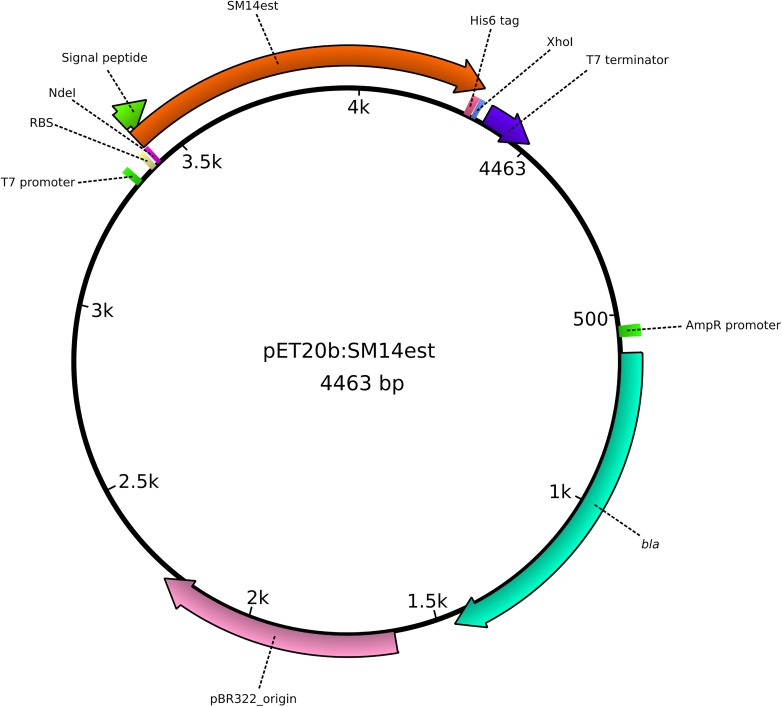
Graphical representation of the pET20b:SM14est plasmid, constructed for the heterologous expression of the SM14est protein in *Escherichia coli*. The insert (SM14est) and other important features of the plasmid are represented and labeled accordingly.

An *E. coli* codon-optimized version of the PETase-like gene was designed, and was synthesized by Eurofins Genomics (Ebersberg, Germany). A 5′ *Nde*I restriction site, a C-terminal His_6_ tag, and a stop codon followed by a 3′ *Xho*I restriction site were added to the gene sequence. Alignment of the nucleotide sequences of the original SM14est gene and the codon-optimized version is shown in Supplementary Figure S1. The synthetic gene was PCR amplified using Phusion Green High-Fidelity DNA Polymerase (Thermo Scientific^TM^) (primers and conditions detailed in Supplementary Table S2), and was subcloned into the pET-20b(+) plasmid (Novagen^®^), resulting in the pET20b:SM14est vector construct (Figure 1), using the NEB^®^ 5-alpha competent *E. coli* (New England Biolabs, Inc., United States) for vector construction and maintenance. The signal peptide of the native protein was predicted using SignalP 5.0 (Almagro Armenteros et al., 2019), and it was maintained in the final construct. The expression vector was then transformed into BL21(DE3) competent *E. coli* (New England Biolabs, Inc., United States) for heterologous protein expression. Confirmation of the insert was performed via (1) restriction digestion of the plasmid DNA with the *Nde*I and *Xho*I restriction enzymes followed by gel electrophoresis analysis and (2) via Sanger sequencing of the insert region of the plasmid, amplified using the T7 standard vector primers (Supplementary Table S2).

## Results and Discussion

### *In silico* Screening of PETase-Like Proteins in *Streptomyces* Genomes and Enzyme Activity Assessment

Previous studies have identified enzymes with plastic-degrading capabilities which have been isolated from different organisms, amongst these were the *Ideonella sakaiensis* strain 201-F6, and isolates from the genera *Thermobifida*, *Thermomonospora*, and *Saccharomonospora* (Wei and Zimmermann, 2017a; Danso et al., 2018; Joo et al., 2018; Kawai et al., 2019). This class of enzymes is commonly referred to as PETase or PETase-like, due to their ability to hydrolyse PET, although at different levels of efficiency. From the bacterial PETase-like class of enzymes discovered to date, the PETase from *Ideonella sakaiensis* strain 201-F6 (referred to from now on as IsPETase) is the one that has received most attention from the scientific community; as it is considered the enzyme which displays the best PET hydrolase activity and substrate specificity which has been discovered thus far (Yoshida et al., 2016; Joo et al., 2018; Kawai et al., 2019). A total of 15 of the most prominent PETase-like enzymes were selected to build the reference data set (Table 1), which was then used to search for potential homologous proteins in the *Streptomyces* genomes data set. As described previously, the *Streptomyces* genomes data set comprised of 52 genome sequences, including 23 terrestrial *Streptomyces* isolates, and 29 isolates derived from the marine environment – 20 of which had previously been isolated by our group and for which genome sequences were available (Supplementary Table S2) (Kennedy et al., 2009; Almeida et al., 2018; Jackson et al., 2018).

**FIGURE 2:**
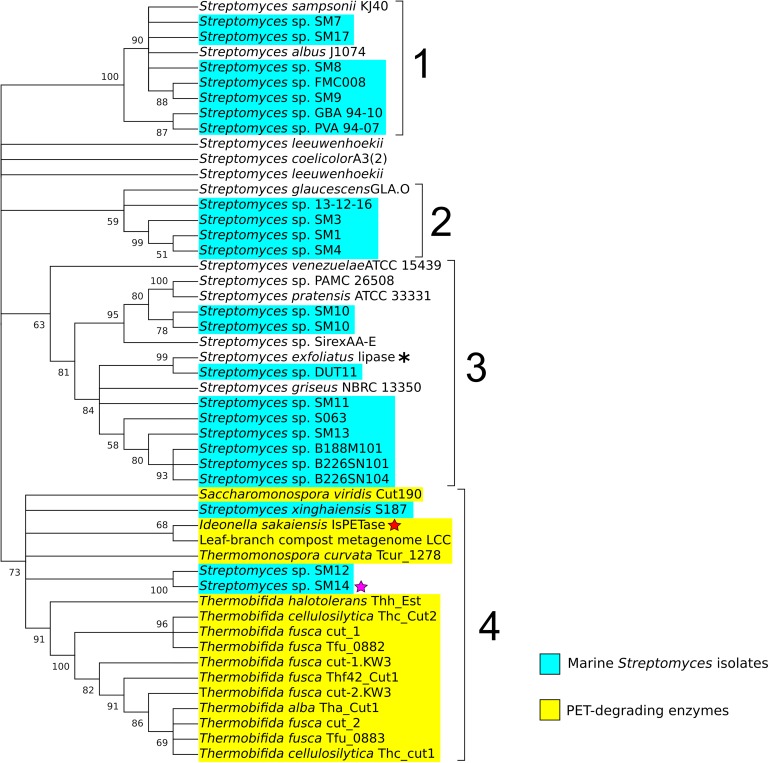
Phylogenetic tree of potential PETase homologs identified in the *Streptomyces* genomes, including terrestrial and marine (highlighted in cyan) *Streptomyces* isolates. The enzymes with known PET-degrading activity are highlighted in yellow. The red star indicates the *Ideonella sakaiensis* IsPETase, the purple star a PETase-like protein from the *Streptomyces* sp. SM14, and the asterisk the lipase from *Streptomyces exfoliatus*. The numbers in the branches indicate the percentage of bootstrap replicates (with a 50% cut-off from 500 replicates) in which the associated taxa clustered together. Repeated strain names indicate the presence of multiple proteins in their respective genomes that shared similarity with the reference PETases proteins.

By applying a sequence similarity search approach using BLASTP (Altschul et al., 1990; Camacho et al., 2009), 34 potential homologous proteins from 32 *Streptomyces* strains were identified, of which the majority were from marine isolates (22 in total). These were then selected for further phylogeny analysis using MEGA X (Kumar et al., 2018). The amino acid sequences of the reference data set, and the *Streptomyces* potential PETase-like homologs were aligned using the Muscle program (Edgar, 2004), and a maximum likelihood phylogenetic tree was generated, with 500 bootstrap replicates. The resulting consensus phylogenetic tree, with a 50% bootstrap value cut-off, comprised of four main clades (Figure 2). Clade number 1 appeared to include *Streptomyces* isolates that were previously reported to share genetic similarity to the type strain *Streptomyces albus* J1074, all of which shared > 99.50% 16S rRNA gene sequence similarity amongst each other (Ian et al., 2014; Zaburannyi et al., 2014). Clade number 2 showed less obvious similarities between all the members of the clade. While it included the strains SM1, SM3 and SM4, that were isolated from the marine sponge *Haliclona simulans* and shared high similarity in their 16S rRNA sequences (>99%) (Kennedy et al., 2009; Jackson et al., 2018), it also included the sponge isolate *Streptomyces* sp. 13-12-16 that shared less 16S rRNA similarity with the aforementioned strains (∼98%), and the soil isolate *Streptomyces glaucescens* strain GLA.O, which in addition to being isolated from a completely different environment, also shares < 99% 16S rRNA similarity with the others in the clade. Clade number 3 appeared to be the most diverse, with *Streptomyces* isolated from varied sources, including: soil, marine sediment, and those isolated from marine sponges, lichens and insects (Ohnishi et al., 2008; Kennedy et al., 2009; Bianchetti et al., 2013; Shin et al., 2013; Xu et al., 2018). Clade 3 also included the lipase from *Streptomyces exfoliatus* (indicated with an asterisk in Figure 2), suggesting that these enzymes are likely to be cutinase-like lipases (Wei et al., 1998; Kawai et al., 2019). Most interesting, however, was clade number 4, which clearly included all of the PETase-like enzymes used in the reference data set, indicating that they may share similar evolutionary processes and history that differentiate them from the other proteins considered in this analysis, which may possibly have led to their ability to degrade synthetic polyesters. It is noteworthy that 3 proteins from marine *Streptomyces* isolates were also included in clade 4, specifically protein sequences from the *Streptomyces* sp. SM12, *Streptomyces* sp. SM14, and *Streptomyces xinghaiensis* S187 isolates, which is a strong indicator that these enzymes may possess plastic-degrading capabilities.

**FIGURE 3:**
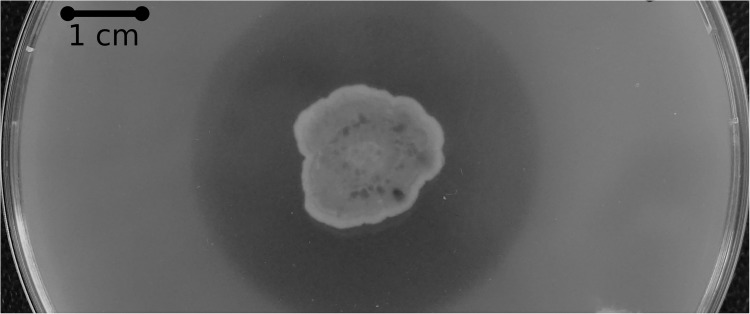
PCL plate clearing assay with the *Streptomyces* sp. SM14 strain incubated at 28°C after 12 days.

Subsequent amino acid sequence analysis showed that the SM12 and SM14 proteins are in fact identical, so additional analysis proceeded with the SM14 strain. The enzyme activity was confirmed with a PCL plate clearing assay – a model substrate to assess PETase and cutinase enzymatic activities (Molitor et al., 2019), in which the SM14 strain was grown in LB medium + 1% PCL emulsion at 28°C for 12 days (Figure 3). The zone of clearing demonstrates the synthetic polyester-degrading capability of the *Streptomyces* sp. SM14 isolate (Figure 3), which is presumably due to the protein identified from the *in silico* screening (Figure 2). Therefore, for the purposes of this study, the SM14 protein will from now on be referred to as SM14est, as it is likely to be a potential polyesterase enzyme. The SM14est gene sequence was deposited in the GenBank database under the accession number BK010828.

In addition, we screened 12 other marine *Streptomyces* isolates from the different clades (clade 1: SM7, SM8, SM9, SM17, and FMC008) (Clade 2: SM1, SM3, SM4) and (Clade 3: SM11, SM13, B188M101, and B226SN104) of the phylogenetic tree identified from the *in silico* screening (Figure 2) using the PCL plate clearing assay. No zones of clearing were however observed following growth of each strain in LB medium + 1% PCL emulsion at 28°C for 12 days (data not shown).

### Protein Structure Analysis

#### Amino Acid Sequence, Conserved Residues, and Domains

**FIGURE 4:**
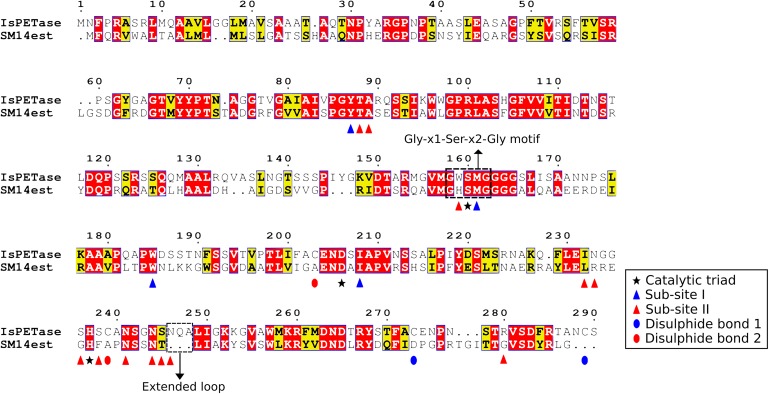
Amino acid sequence alignment of the IsPETase and the SM14est proteins, generated using MEGA X, Muscle, and ESPript 3.0. Identical residues are highlighted in red boxes, and the ones with similar biochemical properties are highlighted in yellow boxes. The serine hydrolase motif Gly-x1-Ser-x2-Gly and the IsPETase extended loop are highlighted in black boxes; the catalytic triad is indicated by a star; the sub-site I and sub-site II are indicated by a blue and a red triangle, respectively. The disulphide bond 1 and disulphide bond 2 are indicated by blue and red ellipses, respectively.

An amino acid sequence comparison between the SM14est and the IsPETase was performed, using the previously described PETase enzyme sites as reference (Joo et al., 2018). The amino acid sequences were aligned using the Muscle algorithm in MEGA X (Edgar, 2004; Kumar et al., 2018), and the alignment and amino acid residues were analyzed in ESPript 3.0 (Figure 4) (Robert and Gouet, 2014). The amino acid alignment showed that 41% of the amino acids in SM14est were identical to the IsPETase, and that an additional 19% of the SM14est amino acids shared similar biochemical properties to the IsPETase. The serine hydrolase motif Gly-x1-Ser-x2-Gly is conserved in both sequences (residues in IsPETase: Gly158-Trp159-Ser160-Met161-Gly162; residues in SM14est: Gly154-His155-Ser156-Met157-Gly158). The catalytic triad is also conserved in both sequences (residues in IsPETase: Ser160, Asp206, His237; and in SM14est: Ser156, Asp202, His234) (Figure 4 and Supplementary Table S3), which is to be expected given that this catalytic triad has been shown to be crucial for enzymatic activity in this class of enzymes. In previous site-direct mutagenesis experiments performed with the IsPETase protein, substitution of any of the residues within the catalytic triad resulted in a complete disruption of the catalysis process (Joo et al., 2018; Liu et al., 2018). One major difference between the IsPETase and our SM14est is that the former possesses two disulphide bonds (the first between Cys273 and Cys289, and the second between Cys203 and Cys239), while the latter has none. Although disulphide bonds are generally related to higher protein thermostability, it has been proposed that the second disulphide bond in IsPETase is connected to its enzymatic activity, since it is positioned in close proximity to the enzyme’s active sites, and substitution of this disulphide bond via site-directed mutagenesis experiments resulted in a drastic decrease in PET hydrolysis (Joo et al., 2018; Liu et al., 2018). However, the requirement of this extra disulphide bond may be exclusive to the IsPETase, since other PETase-like cutinases display PET hydrolase activity and high thermostability without possessing this disulphide bond (Kawai et al., 2019).

Previous molecular docking analysis of the IsPETase, using a four-monohydroxyethyl terephthalate (MHET) molecule that would mimic PET [2-hydroxyethyl-(monohydroxyethyl terephthalate)_4_ or 2-HE(MHET)_4_] as ligand, together with site-directed mutagenesis analysis, suggests that the enzyme possess two sub-sites (Joo et al., 2018). Sub-site I has been proposed to be responsible for the binding of the first MHET moiety, and thus for stabilization of the ligand. Meanwhile, sub-site II has been proposed to be responsible for accommodating the other three MHET moieties, partially leading to the superior PET degradation by the IsPETase in comparison to other PETase-like enzymes. The sub-site I, which consists of 4 residues, is conserved in both IsPETase and SM14est (residues in IsPETase: Tyr87, Met161, Trp185, Ile208; residues in SM14est: Tyr88, Met157, Trp181, Ile204) (Figure 4 and Supplementary Table S3). This implies that both enzymes have a similar mode of binding to the substrate. However, major differences exist between the two sequences in the 12-residue sub-site II region (residues in IsPETase: Thr88, Ala89, Trp159, Ile232, Asn233, Ser236, Ser238, Asn241, Asn244, Ser245, Asn246, Arg280; residues in SM14est: Thr89, Ala90, His155, Leu229, Arg230, Gly233, Phe235, Asn238, Asn241, Thr242, GAP, Gly277) (Figure 4 and Supplementary Table S3). These differences in the sub-site II region in both proteins could lead to different binding affinities to the moieties of the PET polymer. Another important difference between the two protein sequences is in the loop connecting β8 and α6, in which IsPETase appears to possess 3 extra amino acids in comparison to SM14est (Asn246, Gln247, Ala248) (Figure 4). This extended loop has been proposed to be an important structural feature of the IsPETase. When compared to a cutinase from *Thermobifida fusca* KW3, the extended loop seems to provide a conformation that allowed the formation of a continuous cleft on sub-site II – and hence accommodation of the third and fourth MHET moieties. This may represent another structural characteristic that could potentially explain the superior enzymatic activity of IsPETase (Joo et al., 2018).

It has been suggested that the aforementioned protein structural differences between the IsPETase and the cutinase from *Thermobifida fusca* KW3 – namely the absence of two disulphide bonds; differences in the sub-site II residues and the lack of an extended loop – could result in a reduced efficiency in the degradation of PET when compared to the IsPETase. Additionally, it has been proposed that proteins with these characteristics could be classified into the type I category of PETase-like enzymes, which also seems to be the case for the SM14est protein (Joo et al., 2018).

#### Protein Three-Dimensional Structure Prediction and Molecular Docking

**FIGURE 5:**
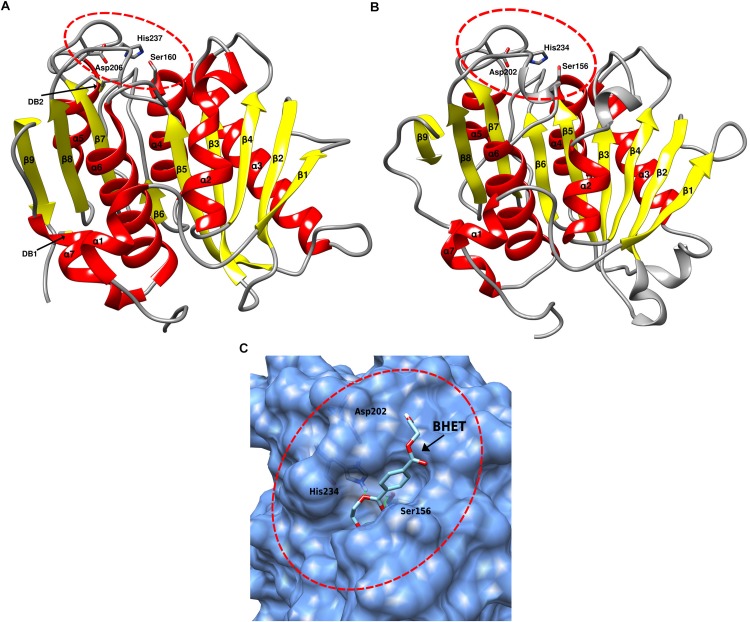
Three-dimensional protein structure comparison and molecular docking. **(A)** IsPETase three-dimensional structure (PDB ID: 5XJH), the catalytic triad (Ser160, Asp206, His237) is circled in red; the two disulphide bonds (DB1 and DB2) are indicated with arrows. **(B)** Predicted three-dimensional structure of the SM14est protein, generated using SWISS-MODEL, with the crystal structure of cutinase 1 from *Thermobifida cellulosilytica* as template (PDB ID: 5LUI). The catalytic triad (Ser156, Asp202, His234) is circled in red. **(C)** Molecular docking simulation performed using AutoDock Vina, with BHET as substrate, indicated with an arrow, detailing the binding pocket, which is circled in red. The catalytic triad residues are shown as sticks. Structures were analyzed and rendered using the UCSF Chimera software.

The function and stability of proteins is closely linked to its conformation, or folded/native state (Lumry and Eyring, 1954; England and Haran, 2011; Balcão and Vila, 2015). To provide further insights into the potential functionality and conformation of the SM14est protein, a three-dimensional structure of the protein was *in silico* predicted using SWISS-MODEL (Benkert et al., 2011; Bertoni et al., 2017; Bienert et al., 2017; Waterhouse et al., 2018). The cutinase 1 (Thc_Cut1) from *Thermobifida cellulosilytica* (PDB ID: 5LUI) was used (Ribitsch et al., 2017) as a template for the model prediction, which generated a model with a GMQE score of 0.76 and a QMEAN Z-score of −1.76, indicating a reliable predicted model (Figure 5B). When compared to the structure of the IsPETase (Figure 5A), the predicted structure of the SM14est shows many similarities, with both belonging to the α/β hydrolase superfamily (Ollis et al., 1992; Nardini and Dijkstra, 1999; Hotelier et al., 2004), displaying a similar arrangement of 9 β-sheets and 7 α-helixes (Figure 5). The arrangement of the catalytic triad residues is also quite similar, as highlighted in Figures 5A,B, which may partially explain the synthetic polyester-degrading activity of these enzymes. With respect to observed potential differences between the two protein structures, the most striking differences – as previously shown in the amino acid sequence comparison, were the lack of disulphide bonds in the SM14est, and the absence of an extended loop between β8 and α6. The latter, as previously mentioned, has been proposed to be linked to proper accommodation of the MHET moieties constituting the PET polymer, and therefore the superior enzymatic activity of the IsPETase (Joo et al., 2018).

Molecular docking experiments were then performed, to analyze the likelihood of the SM14est enzyme possessing the capacity to bind plastics as substrates using an *in silico*-based approach; as well as determining the most probable binding mode of the protein to the ligand. To this end, the AutoDock Vina program was used for the protein-ligand molecular docking simulations (Trott and Olson, 2009), using the previously predicted SM14est structure and the BHET molecule as the ligand. The molecular docking experiment generated six binding modes with energy ≤ -5.0 kcal/mol, similar to the binding modes energy values that had previously been described in similar molecular docking experiment performed with IsPETase and BHET (Liu et al., 2018). The binding modes were analyzed in more detail using the UCSF Chimera software (Pettersen et al., 2004), and the best mode with the lowest binding energy is represented in Figure 5C, which highlights the binding pocket of the enzyme, the catalytic triad, and the proposed binding mode of the BHET molecule. The protein structure and molecular docking analyses results further emphasize the potential of the SM14est to degrade plastics, and highlight the structural features that may facilitate this enzymatic activity.

### Heterologous Expression of SM14est

**FIGURE 6:**
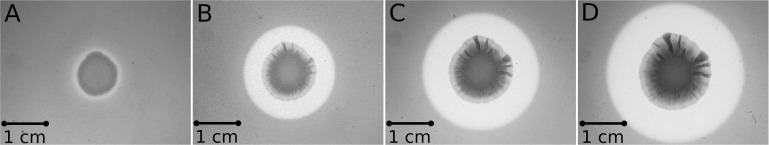
PCL plate clearing assay with the *E. coli* BL21(DE3)(pET20b:SM14est) heterologous host, incubated at 28°C after **(A)** 1 day; **(B)** 2 days; **(C)** 3 days; **(D)** 4 days.

To determine whether the SM14*est* gene does possess polyesterase activity, the gene was firstly codon-optimized for *E. coli* to facilitate heterologous expression in this host. The gene was then cloned into the expression vector pET-20b(+), generating the construct pET20b:SM14est, in which the native signal peptide sequence was maintained (Figure 1). The construct was then transformed into *E. coli* BL21(DE3) and transformants were tested for activity by performing a PCL plate clearing assay (Figure 6). A halo of clearing, which is indicative of PCL degradation, was observed following 1 day of incubation (Figure 6A), with the activity subsequently increasing after 2, 3, and 4 days (Figures 6B–D, respectively). The negative control with *E. coli* BL21(DE3) containing the pET-20b(+) plasmid without the insert did not show any PCL-degrading activity (data not shown).

Another interesting observation was that the *E. coli* host successfully exported the heterologously expressed SM14est enzyme, when the native *Streptomyces* sp. SM14 signal peptide sequence was present in the expression construct (Figures 1, 6). The signal peptide sequence was predicted to consist of the first 25 amino acids of the protein sequence, with a cleavage site probability of 0.9316, and to belong to the general secretory (Sec) pathway, with a likelihood value of 0.9608. In heterologous protein expression systems involving *E. coli*, successful secretion and maintenance of the native protein confirmation can sometimes be challenging, and in this case potentially so, due to the fact the SM14est protein originates from such a distant host, i.e., a *Streptomyces* isolate (Freudl, 2018). Several *Streptomyces* genes encoding different enzymes have previously been heterologously expressed in *E. coli* (Spasic et al., 2018) – including a xylanase from *S. mexicanus* HY-14 JQ943651 (Kim et al., 2014); a laccase from *S. coelicolor* (Sherif et al., 2013); a protease from *S. koyangensis* (Ben Elhoul et al., 2015); a glucose isomerase from *Streptomyces* sp. SK (Ben Hlima et al., 2013); and an esterase from *S. lividans* (Wang et al., 2016). In these cases however, unlike with the heterologous expression of the SM14est in *E. coli*, the native signal sequence was not employed in the expression constructs.

A number of different signal peptide sequences have previously been employed to ensure the secretion of PETase and PETase-like enzymes from *E. coli*. A PET carboxylesterase from *Thermobifida fusca* has been expressed and secreted from *E. coli* using a pelB leader sequence (Oeser et al., 2010), while a PET hydrolase has also previously been expressed and secreted from *Bacillus subtilis* using a native PETase signal peptide (SP_PETase_) (Huang et al., 2018). In addition, a Sec-dependent signal sequence from *E. coli* has also recently been used to express the IsPETase, resulting in the production of the extracellular enzyme using *E. coli* (Seo et al., 2019). The successful secretion of heterologously expressed PETases is important not only for their subsequent purification, and biochemical characterization (Han et al., 2017; Joo et al., 2018; Liu et al., 2018), but also for their potential development as recombinant PET-degrading microbes with utility in environmental remediation strategies. Therefore, it is interesting to note that we were able to efficiently heterologous express the PETase-like SM14est enzyme from *Streptomyces* sp. in *E. coli*, without the requirement to change its native signal peptide sequence, with extracellular synthetic polyester-degrading activity being observed in a PCL plate clearing assay. To our knowledge this is the first report of a PETase-like enzyme being identified in a marine sponge-derived *Streptomyces* sp. isolate, and we believe that the PETase-like SM14est enzyme will help provide further insights into our current knowledge of this important class of synthetic polyester-degrading enzymes.

## Conclusion

Plastics such as the PET have been commonly used in storage materials and in synthetic fabrics, and their resistance to biodegradation has resulted in their accumulation in terrestrial and marine ecosystems at an alarming rate. In an attempt to alleviate this problem, much recent scientific interest has focused on the enzymatic hydrolysis of these types of synthetic polyesters, including PET. While a number of PETase and PETase-like enzymes have been identified and biochemically characterized, there is still much to be learned about this class of enzymes. In addition, more information on their structure, activity, and how widespread they are distributed in nature is required; and if they can ultimately be improved using genetic and protein engineering and applied in bioremediation strategies on an industrial scale.

Although the *Streptomyces* genus is well-studied with respect to the production of bioactive compounds, less is known about their potential to produce enzymes with synthetic polyester-degradation activities. In this study, based on an *in silico* screening approach, we were able to identify a PETase-like enzyme, namely SM14est, with synthetic polyester-degrading activity, which was isolated from the marine sponge-derived strain *Streptomyces* sp. SM14, with enzyme activity being confirmed *in vitro* with the heterologous expression of the protein in *E. coli* using PCL plate clearing assays. Importantly, an active heterologously expressed SM14est protein was secreted from *E. coli* with the native *Streptomyces* SM14est signal peptide sequence. This will facilitate not only the future biochemical characterization of the protein, but also its potential utility in other bioremediation-based applications targeting synthetic polyesters.

## Data Availability Statement

The raw data supporting the conclusions of this manuscript will be made available by the authors, without undue reservation, to any qualified researcher.

## Author Contributions

EA, AC, SJ, and AD conceived and designed the experiments. EA and AC performed the experiments. EA, AC, and AD analyzed the data. SJ and AD contributed reagents, materials, and analysis tools. EA and AD wrote the manuscript.

## Conflict of Interest

The authors declare that the research was conducted in the absence of any commercial or financial relationships that could be construed as a potential conflict of interest.

## Footnotes

1 http://mgltools.scripps.edu/
