# Supplementary material for: In silico Screening and Heterologous Expression of a Polyethylene Terephthalate Hydrolase (PETase)-Like Enzyme (SM14est) With Polycaprolactone (PCL)-Degrading Activity, From the Marine Sponge-Derived Strain Streptomyces sp. SM14
Source: Front Microbiol. 2019 Oct 1;10:2187. doi: 10.3389/fmicb.2019.02187 (PMC6779837; doi:10.3389/fmicb.2019.02187)
Supplement: Supplementary file 1 [file Image_1.pdf]

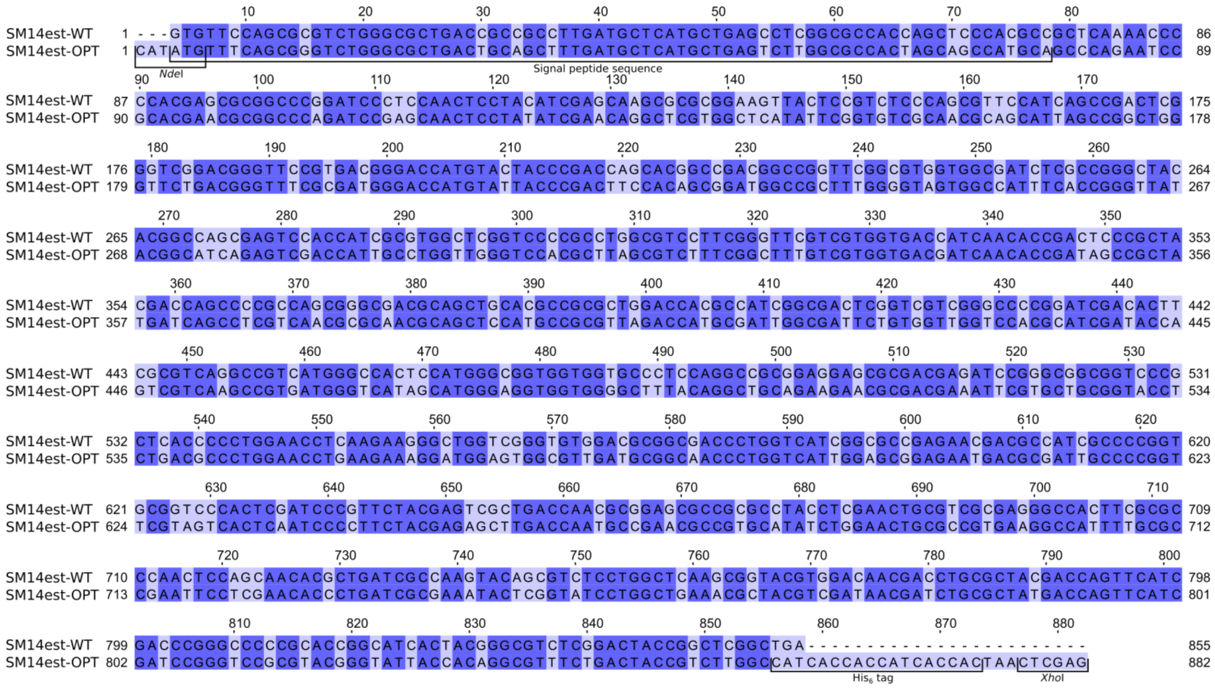

Figure S1: Nucleotide sequences alignment of the native SM14est protein codon usage (SM14est-WT) and the *E. coli* codon optimised synthetic version of the gene (SM14est-OPT). Darker boxes indicate identical nucleotides, while the lighter boxes indicate differences. Features such as the signal peptide sequence, and the engineered His_6 tag and restriction sites (*Nde*I and *Xho*I) are indicated accordingly.
