# Supplementary material for: In silico Screening and Heterologous Expression of a Polyethylene Terephthalate Hydrolase (PETase)-Like Enzyme (SM14est) With Polycaprolactone (PCL)-Degrading Activity, From the Marine Sponge-Derived Strain Streptomyces sp. SM14
Source: Front Microbiol. 2019 Oct 1;10:2187. doi: 10.3389/fmicb.2019.02187 (PMC6779837; doi:10.3389/fmicb.2019.02187)
Supplement: Supplementary file 2 [file Table_1.pdf]

Table S1: *Streptomyces* genome sequences employed in this study, obtained from the GenBank database when indicated. The isolation source is discriminated as marine and non-marine environment.

Organism name	GenBank accession number	Source
Streptomyces alboflavus strain MDJK44	CP021748.1	Non-marine
Streptomyces albus strain BK3-25	NZ_CP016825.1	Non-marine
Streptomyces albus strain DSM 41398	NZ_CP010519.1	Non-marine
Streptomyces albus J1074	NC_020990.1	Non-marine
Streptomyces globisporus C-1027	CP013738.1	Non-marine
Streptomyces griseus subsp. *griseus* NBRC 13350	NC_010572.1	Non-marine
Streptomyces sp. PAMC26508	NC_021055.1	Non-marine
Streptomyces pratensis ATCC 33331	CP002475.1	Non-marine
Streptomyces sampsonii strain KJ40	NZ_CP016824.1	Non-marine
Streptomyces avermitilis MA-4680	BA000030.4	Non-marine
Streptomyces bingchenggensis BCW-1	CP002047.1	Non-marine
Streptomyces cattleya DSM 46488	CP003219.1	Non-marine
Streptomyces coelicolor A3(2)	AL645882.2	Non-marine
Streptomyces collinus Tu 365	CP006259.1	Non-marine
Streptomyces glaucescens strain GLA.O	CP009438.1	Non-marine
Streptomyces sp. SirexAA-E	NC_015953.1	Non-marine
Streptomyces leeuwenhoekii	LN831790.1	Non-marine
Streptomyces lincolnensis strain NRRL 2936	CP016438.1	Non-marine

Streptomyces noursei ATCC 11455	CP011533.1	Non-marine
Streptomyces rimosus subsp. *rimosus* strain NRRL ISP-5260	NYR01000001.1	Non-marine
Streptomyces scabiei 87.22	FN554889.1	Non-marine
Streptomyces venezuelae strain ATCC 15439	CP013129.1	Non-marine
Streptomyces vietnamensis strain GIM4.0001	CP010407.1	Non-marine
Streptomyces sp. 13-12-16	NCTE01000967.1	Marine
Streptomyces sp. CNQ-509	CP011492.1	Marine
Streptomyces sp. DUT11	CP025511.1	Marine
Streptomyces sp. GBA 94-10	CM002271.1	Marine
Streptomyces sp. PVA 94-07	CM002273.1	Marine
Streptomyces sp. RV15	KQ949075.1	Marine
Streptomyces sp. S063	CP021707.1	Marine
Streptomyces sp. SCSIO 03032	CP021121.1	Marine
Streptomyces xinghaiensis S187	CP023202.1	Marine
Streptomyces sp. B188M101	GCA_002910985.1	Marine
Streptomyces sp. B226SN101	GCA_002910935.1	Marine
Streptomyces sp. B226SN104	Not deposited	Marine
Streptomyces sp. FMC008	Not deposited	Marine
Streptomyces sp. SM1	GCA_002910825.1	Marine
Streptomyces sp. SM3	Not deposited	Marine
Streptomyces sp. SM4	Not deposited	Marine

Streptomyces sp. SM5	GCA_002910895.1	Marine
Streptomyces sp. SM6	Not deposited	Marine
Streptomyces sp. SM7	Not deposited	Marine
Streptomyces sp. SM8	GCA_000299175.2	Marine
Streptomyces sp. SM9	GCA_002910795.1	Marine
Streptomyces sp. SM10	GCA_002910915.1	Marine
Streptomyces sp. SM11	GCA_002910905.1	Marine
Streptomyces sp. SM12	GCA_002910855.1	Marine
Streptomyces sp. SM13	GCA_002910875.1	Marine
Streptomyces sp. SM14	GCA_002910755.1	Marine
Streptomyces sp. SM17	GCA_002910725.2	Marine
Streptomyces sp. SM18	GCA_002910775.2	Marine
Streptomyces sp. SM19	Not deposited	Marine
