# Supplementary material for: In silico Screening and Heterologous Expression of a Polyethylene Terephthalate Hydrolase (PETase)-Like Enzyme (SM14est) With Polycaprolactone (PCL)-Degrading Activity, From the Marine Sponge-Derived Strain Streptomyces sp. SM14
Source: Front Microbiol. 2019 Oct 1;10:2187. doi: 10.3389/fmicb.2019.02187 (PMC6779837; doi:10.3389/fmicb.2019.02187)
Supplement: Supplementary file 3 [file Table_2.pdf]

Table S2: Primers and respective PCR amplification conditions employed in this study. The primer pair SM14est_fw and SM14est_rev was employed to subclone the synthetic SM14est gene sequence into the pET-20b(+) vector. The primer pair T7 and T7_term (T7 standard primers) was employed to confirm the insert in the construct pET20b:SM14est.

<table>
<tr><th>Primer name</th><th>Primer sequence</th><th>PCR conditions</th></tr>
<tr><td>SM14est_fw</td><td>5’-AAAAACATATGTTTCAGCGGGTCTGGGCGCTG-3’</td><td rowspan="2">Step 1: Incubate at $98^{o}C$ for 30 s
Step 2: Incubate at $98^{o}C$ for 10 s
Step 3: Incubate at $72^{o}C$ for 30 s
Step 4: Cycle to step 2 for 35 times
Step 5: Incubate at $72^{o}C$ for 10 min</td></tr>
<tr><td>SM14est_rev</td><td>5’-AAAAACTCGAGTTAGTGGTGATGGTGGTGATGGC-3’</td></tr>
<tr><td>T7</td><td>5’-TAATACGACTCACTATAGG-3’</td><td rowspan="2">Step 1: Incubate at $98^{o}C$ for 30 s
Step 2: Incubate at $98^{o}C$ for 10 s
Step 3: Incubate at $53^{o}C$ for 30 s
Step 4: Incubate at $72^{o}C$ for 15 s
Step 5: Cycle to step 2 for 35 times
Step 6: Incubate at $72^{o}C$ for 10 min</td></tr>
<tr><td>T7_term</td><td>5’-CTAGTTATTGCTCAGCGGT-3’</td></tr>
</table>
