# Supplementary material for: In silico Screening and Heterologous Expression of a Polyethylene Terephthalate Hydrolase (PETase)-Like Enzyme (SM14est) With Polycaprolactone (PCL)-Degrading Activity, From the Marine Sponge-Derived Strain Streptomyces sp. SM14
Source: Front Microbiol. 2019 Oct 1;10:2187. doi: 10.3389/fmicb.2019.02187 (PMC6779837; doi:10.3389/fmicb.2019.02187)
Supplement: Supplementary file 4 [file Table_3.pdf]

Table S3: Comparison of residues constituting the catalytic triad, sub-site I, and sub-site II in IsPETase and SM14est. Residue substitutions in SM14est are indicated with an asterisk, and those substitutions involving amino acids with similar biochemical properties are indicated with two asterisks.

	Residue in IsPETase	Residue in SM14est
**Catalytic triad**	Ser160	Ser156
	Asp206	Asp202
	His237	His234
**Sub-site I**	Tyr87	Tyr88
	Met161	Met157
	Trp185	Trp181
	Ile208	Ile204
**Sub-site II**	Thr88	Thr89
	Ala89	Ala90
	Trp159	His155*
	Ile232	Leu229**
	Asn233	Arg230*
	Ser236	Gly233*
	Ser238	Phe235*
	Asn241	Asn238
	Asn244	Asn241
	Ser245	Thr242**
	Asn246	-*

	Arg280	Gly277*
